# Photoelectrochemical Performance of Quantum dot-Sensitized TiO_2_ Nanotube Arrays: a Study of Surface Modification by Atomic Layer Deposition Coating

**DOI:** 10.1186/s11671-017-2036-6

**Published:** 2017-04-07

**Authors:** Quan Zhou, Junchen Zhou, Min Zeng, Guizhen Wang, Yongjun Chen, Shiwei Lin

**Affiliations:** 1 State Key Laboratory of Marine Resource Utilization in South China Sea, Hainan University, Haikou, 570228 People’s Republic of China; 2 College of Materials and Chemical Engineering, Hainan University, Haikou, 570228 People’s Republic of China

**Keywords:** TiO_2_ nanotube arrays, Quantum dots, Surface modification, Atomic layer deposition, Photoelectrochemical performance

## Abstract

Although CdS and PbS quantum dot-sensitized TiO_2_ nanotube arrays (TNTAs/QDs) show photocatalytic activity in the visible-light region, the low internal quantum efficiency and the slow interfacial hole transfer rate limit their applications. This work modified the surface of the TNTAs/QDs photoelectrodes with metal-oxide overlayers by atomic layer deposition (ALD), such as coating Al_2_O_3_, TiO_2_, and ZnO. The ALD deposition of all these overlayers can apparently enhance the photoelectrochemical performance of the TNTAs/QDs. Under simulated solar illumination, the maximum photocurrent densities of the TNTAs/QDs with 10 cycles ZnO, 25 cycles TiO_2_, and 30 cycles Al_2_O_3_ overlayers are 5.0, 4.3, and 5.6 mA/cm^2^ at 1.0 V (vs. SCE), respectively. The photoelectrode with Al_2_O_3_ overlayer coating presents the superior performance, whose photocurrent density is 37 times and 1.6 times higher than those of the TNTAs and TNTAs/QDs, respectively. Systematic examination of the effects of various metal-oxide overlayers on the photoelectrochemical performance indicates that the enhancement by TiO_2_ and ZnO overcoatings can only ascribed to the decrease of the interfacial charge transfer impedance, besides which Al_2_O_3_ coating can passivate the surface states and facilitate the charge transfer kinetics. These results could be helpful to develop high-performance photoelectrodes in the photoelectrochemical applications.

## Background

TiO_2_ is a kind of inorganic semiconductor materials with excellent photocatalytic activity, non-toxicity, and good stability. It is widely used in the fields of water splitting, photocatalysis, solar cells, lithium ion battery, etc. In 1972, Honda and Fujishima have achieved water splitting using TiO_2_ photoanode under xenon lamp irradiation [1]. Different architectures of TiO_2_ nanomaterials have been fabricated, such as nanoparticles [2], nanowires [3], nanotubes [4], nanosheets [5], nanoflowers [6], hollow spheres [7] etc. Especially the nanotube arrays have been widely studied with excellent optical excitation and charge transfer properties. However, TiO_2_ cannot effectively use the visible light because of its wide bandgap (∼3.2 eV). Transition metals [8] or nonmetals [9] doping can reduce the bandgap of TiO_2_, but the dopants can easily become the electron-hole recombination centers [10]. The other method is using narrow-bandgap materials such as quantum dots (QDs) to sensitize TiO_2_ and enhance their photocatalytic activity in the visible-light region since the photogenerated charge can quickly separate and transfer between different semiconductors [11, 12]. CdS [13], CdSe [14], PbS [15], and PbSe [16] quantum dots can respond to the visible light with a tunable bandgap [17]. In recent years, co-sensitized TiO_2_ with quantum dots such as CdSe/CdTe [18], CdS/CdSe [19], and PbS/CdS [20] have been reported. However, the efficiency of quantum dot-sensitized TiO_2_ photoelectrodes is still at a lower level. The main reasons are the serious optical etching and the large number of defects in the surface of the quantum dots which will lead to the formation of charge recombination centers.

Surface modification by atomic layer deposition (ALD) is an advanced method to prepare metal-oxide layers on the electrodes to improve their performance. Al_2_O_3_, TiO_2_, and ZnO are three kinds of common metal oxides which could be prepared by ALD for surface modification. The process is that two precursors are separately introduced into the reaction chamber by alternation pulse, and the chemical adsorption and surface reaction will occur on the sample [21]. Compared with traditional deposition methods, ALD can prepare uniform and controllable thin film. Metal oxides prepared by ALD technique have been used to passivate surface defects of semiconductor materials in the fields of photocatalysis and water splitting. For photoelectrochemical water splitting, ALD technique has been employed and showed promising results for Al_2_O_3_/TiO_2_ [22], TiO_2_/TiO_2_ nanorod arrays [23], ZnO/TiO_2_ [24, 25], and Co_3_O_4_/TiO_2_ [26]. In the previous reports, however, such metal-oxide layers were mostly used as an energy barrier between quantum dots sensitizer and semiconductor electrodes to suppress charge recombination and improve the incorporated amounts of QDs. There are still few systematic studies on the effects of metal-oxide overlayers on the photoelectrochemical performance of the QD-sensitized photoelectrodes, and even no comprehensive comparison of Al_2_O_3_, TiO_2_, and ZnO has been reported ﻿so far. However, such information is not only significant to understand the various underlying mechanisms of the different overcoatings but also valuable for proper selection of metal-oxide overlayer to improve the photoelectric properties and for rational design and optimization of high-performance photoelectrodes.

We report here the improving photoelectrochemical performance of quantum dot-co-sensitized TiO_2_ nanotube arrays through surface modification by ALD coating various metal-oxide overlayers. The CdS and PbS quantum dot-co-sensitized TNTA photoelectrode shows a significant enhancement in the visible-light absorption range between 400 and 600 nm. Three kinds of metal-oxide overlayers, Al_2_O_3_, TiO_2_, and ZnO, have been uniformly coated throughout the surface of the photoelectrodes by ALD. Their photoelectrochemical performance has been studied by linear sweep voltammetry, electrochemical impedance spectroscopy, and Mott-Schottky analysis. The systematic examination of the role of various metal-oxide overlayers reveals the underlying mechanism of the enhancement. The results show that ALD deposition of metal-oxide overlayers is a promising and facile method to improve the photoelectric properties of the semiconductor electrodes.

## Methods

### Preparation of TiO_2_ Nanotube Arrays

Prior to electrochemical anodization, Ti foils with a thickness of 0.5 mm and purity of 99.5% were chemical polished and ultrasonically cleaned in acetone, ethanol, and deionized water (DI) for 15 min successively. TNTAs were prepared by anodic oxidation method in a two-electrode system with titanium plates as working electrode and stainless steel sheet as counter electrode. The electrolyte was 0.27 M NH4F solution, and the solvent was a mixture of glycerin and DI water with the volume ratio of 1:1. The anodic oxidation was conducted at a biased of 25 V for 5 h at room temperature [27]. After the oxidation, the TNTAs obtained were soaked in ethanol for 3 min ultrasonic cleaning in order to remove the white floc surface. In order to convert amorphous structures to the crystalline phase, the samples were annealed at 450 °C for 3 h and 45 min with a heating rate of 2 °C/min, and then heat cooled to room temperature in the furnace.

### Preparation of CdS/PbS Co-sensitized TiO_2_ Nanotube Arrays

PbS and CdS quantum dots were loaded over crystallized TiO_2_ nanotube arrays by the successive ionic layer adsorption and reaction (SILAR) method. The specific steps are as follows: The precursor solutions were 0.02 M Pb (NO_3_)_2_ methanol solution and 0.02 M Na_2_S solution of methanol and deionized water (*V*:*V* = 1:1). TNTAs were first dipped in 0.02 M Pb (NO_3_)_2_ methanol solution for 15 s, and then keep for 1 min 45 s, rinsed with methanol, dipped in 0.02 M Na_2_S solution for 15 s, and then keep for 1 min 45 s rinsed with methanol; two procedures were termed as one deposition cycle of PbS quantum dots and then repeated 5 times. Secondly, the samples were sensitized with CdS quantum dots immediately. Similar to the procedure above, the deposition cycles of CdS quantum dots were 5 as well. The precursor solutions were 0.05 M Cd (NO_3_)_2_ ethanol solution and 0.05 M Na_2_S solution (volume ratio of methanol and DI water is 1:1). The sample is referred to as TNTAs/QDs [28]. Finally, the sample was covered with 2 cycles ZnS protecting layer to reduce surface recombination [28, 29].

### Preparation of TiO_2_ Co-sensitized CdS/PbS Nanotube Arrays Modified by ALD Metal Oxide Overlayers

ALD method was then applied for metal-oxide overlayer coating. In order to guarantee that the photogenerated electrons can tunnel through the overlayer and the results are comparable, the overlayer thickness is kept about 1.5 nm. This requires the overlayer deposition with 30 cycles Al_2_O_3_ (0.5 Å/cycle), 25 cycles TiO_2_ (0.6 Å/cycle), and 10 cycles ZnO (1.5 Å/cycle), respectively. The precursor was dissolved in 1 M heptane in solution, the reactor temperature is 150 °C, and N_2_ as carrier gas and washing gas. For Al_2_O_3_ deposition, three methyl aluminum (trimethylaluminum, TMA) was used as precursor. The specific steps are as follows. TNTAs/QDs samples were first put in the chamber. An ALD cycle of Al_2_O_3_ deposition consisted of a 0.02 s pulse of TMA, 5 s exposure to TMA, 15 s purge with N_2_ and then 0.05 s pulse of H_2_O, 5 s exposure to H_2_O, final 15 s purge with N_2,_ thus completing a cycle of Al_2_O_3_ deposition [30]. An ALD cycle of TiO_2_ deposition and ZnO deposition were similar to the above procedures but selected diethyl zinc (DEZ) and titanium (IV) isopropoxide as their precursors, respectively [31]. In order to minimize the effect of surface area variation, we carefully controlled the process condition for preparing the quantum dot-sensitized TiO_2_ nanotube arrays and kept the thickness of the passivation layer deposited as about 1.5 nm.

### Characterization and Photoelectrochemical Performance Testing

The surface morphology and microstructure of the samples were observed by using scanning electron microscopy (FESEM, Hitachi, S4800) and transmission electron microscopy (TEM, JEM 2100). Energy dispersive X-ray spectrometer (EDS) accompanying FESEM was used to analyze sample element. X-ray diffraction (XRD, Bruker D8) with Cu Ka radiation was used to analyze the crystal structure of the samples. The optical absorption properties of the samples were recorded by UV-Vis spectrophotometer (UV, Purkinje TU-1901).

The photoelectric properties of the samples were tested by electrochemical workstation (Zennium Zahner) in a standard three-electrode setup with the prepared sample as the working electrode with a specific exposure area of 1 cm^2^. A double salt bridge saturated calomel electrode (SCE) and a Pt wire were the reference and the counter electrodes, respectively. The electrolyte was a aqueous solution mixed with 0.1 M Na_2_S and 0.2 M Na_2_SO_3_ with a volume ratio of 1:1 (pH = 12.2). The voltage scan rate of linear sweep voltammetry (LSV) test was 10 mV/s, and the excitation light source was a 500 W xenon lamp with the wavelength range from 350 to 1100 nm and calibrated through an AM 1.5 filter and PC-2 solar radiation recorder (AM 1.5G, 100 mW/cm^2^). LED lamp was used as the excitation light source of the transient photocurrent test with electrochemical workstation (wavelength 565 + 112 nm; light intensity 80 mW/cm^2^), while every 25 s for a range of switch lamp to achieve light and dark conditions.

Electrochemical impedance spectroscopy (EIS) was examined under open-circuit voltage and simulated solar light condition. The amplitude was 10 mV and the frequency range was from 0.1 Hz to 100 KHz. Mott-Schottky analysis was performed in the dark condition, and the voltage range is from −1 to 0.9 V. The scanning rate was 30 mV/s, and the frequency and amplitude perturbation were 1 kHz and 10 mV.

## Results and Discussion

**Fig. 1:**
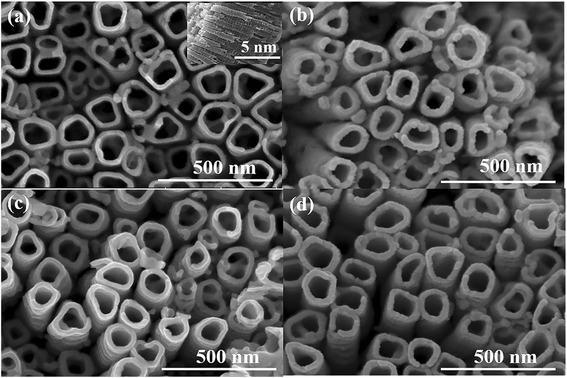
SEM images of TNTAs/QDs with various ALD cycles overlayer: **a** 0 cycles, **b** 30 cycles Al_2_O_3_, **c** 10 cycles ZnO, and **d** 25 cycles TiO_2_. The *inset* in **a** is a cross-sectional view of the well-aligned quantum dot-sensitized TiO_2_ nanotube arrays without any overlayer

Figure 1 depicts the quantum dot-sensitized TiO_2_ nanotube arrays with different metal oxide overlayers. The structure of TiO_2_ nanotube arrays is highly ordered on Ti foils with smooth surface and an average tube inner diameter of 110 nm as shown in Fig. 1a. The inset in Fig. 1a is a cross-sectional view of the quantum dot-sensitized TiO_2_ nanotube arrays. The length of the nanotubes is about 1.7 μm and the wall thickness is about 23 nm. The size of the quantum dots is too small to be observed by SEM. The inner diameters of TiO_2_ nanotubes decrease after oxide layer coating by ALD (Fig. 1b–d), indicating conformal overlayer deposition along the high-aspect ratio nanotubes.

**Fig. 2:**
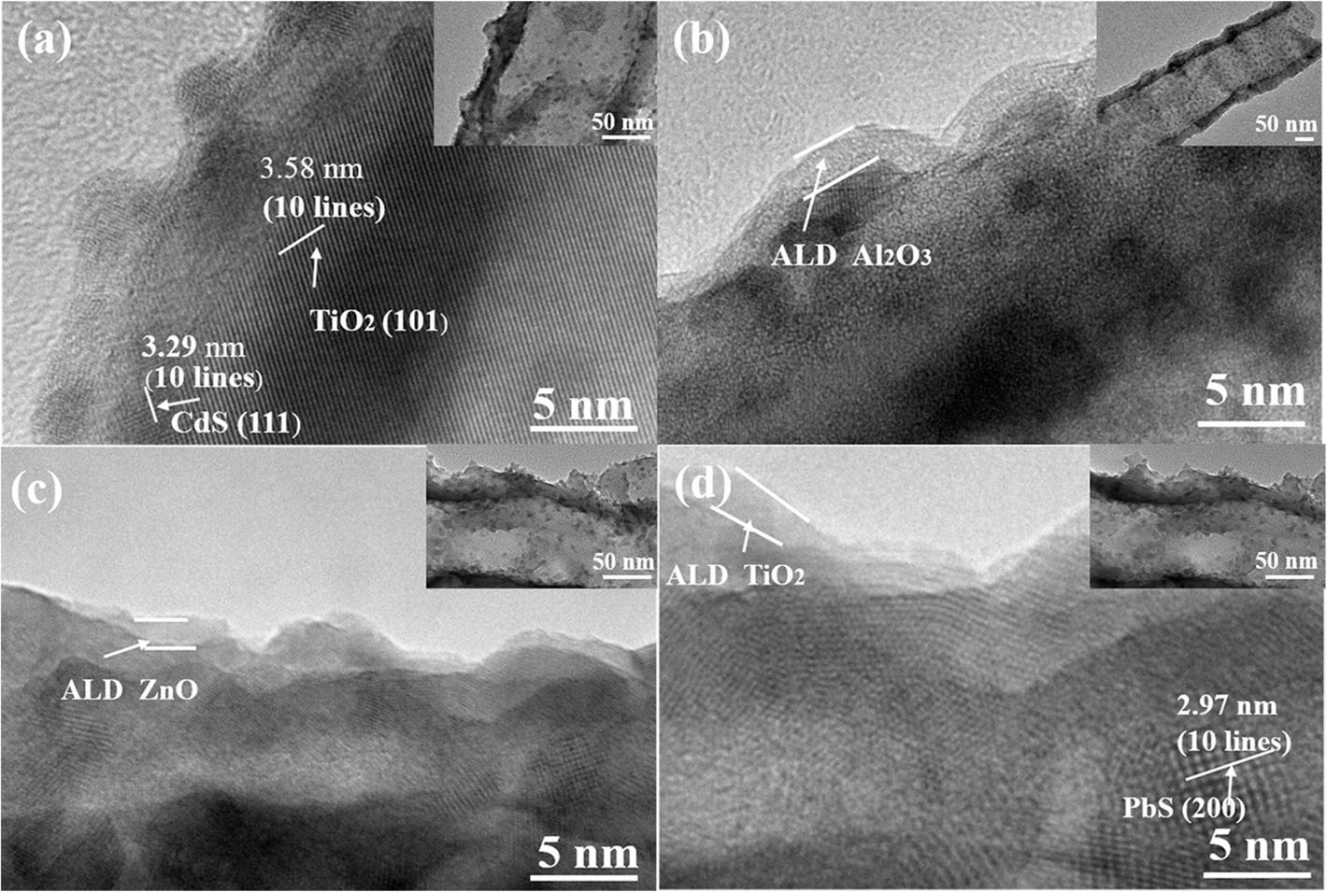
TEM images of TNTAs/QDs with various ALD cycles overlayer: **a** 0 cycles, **b** 30 cycles Al_2_O_3_, **c** 10 cycles ZnO, and **d** 25 cycles TiO_2_. The *insets* are the corresponding low-resolution TEM images

Figure 2 is the high-resolution TEM (HRTEM) images of the TNTAs/QDs both before and after the metal-oxide layer deposition. The illustration in the insets is the corresponding low-resolution TEM images. It can be seen that the quantum dots are evenly distributed on the TiO_2_ nanotubes with a particle size of approximately 8 nm. It can be seen from Fig. 2b–d that a smooth, uniform, and light-color layer with thickness of approximately 1.5 ± 0.5 nm is wrapped outside the TNTAs/QDs. The thin layer are Al_2_O_3_, ZnO, and TiO_2_ coating, respectively. In Fig. 2a, the spacing of 3.29 and 3.58 Å, respectively, correspond to the (111) lattice plane of the cubic phase CdS (JCPDS No. 89-0440) and the (101) lattice plane of the TiO_2_ anatase type (JCPDS 21-1272); in Fig. 2d, the lattice spacing of 2.97 Å correspond to the (200) lattice plane of the cubic phase PbS.

**Fig. 3:**
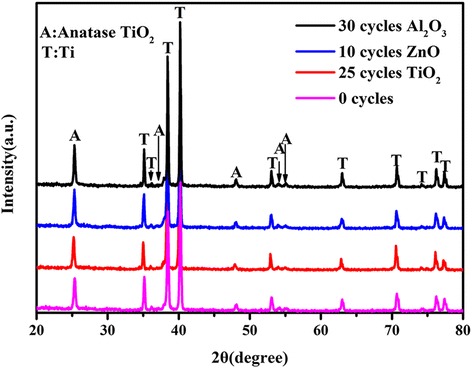
XRD patterns of the TNTAs/QDs, TNTAs/QDs/10 cycles ZnO, TNTAs/QDs/25 cycles TiO_2_, and TNTAs/QDs/30 cycles Al_2_O_3_ electrodes

**Fig. 4:**
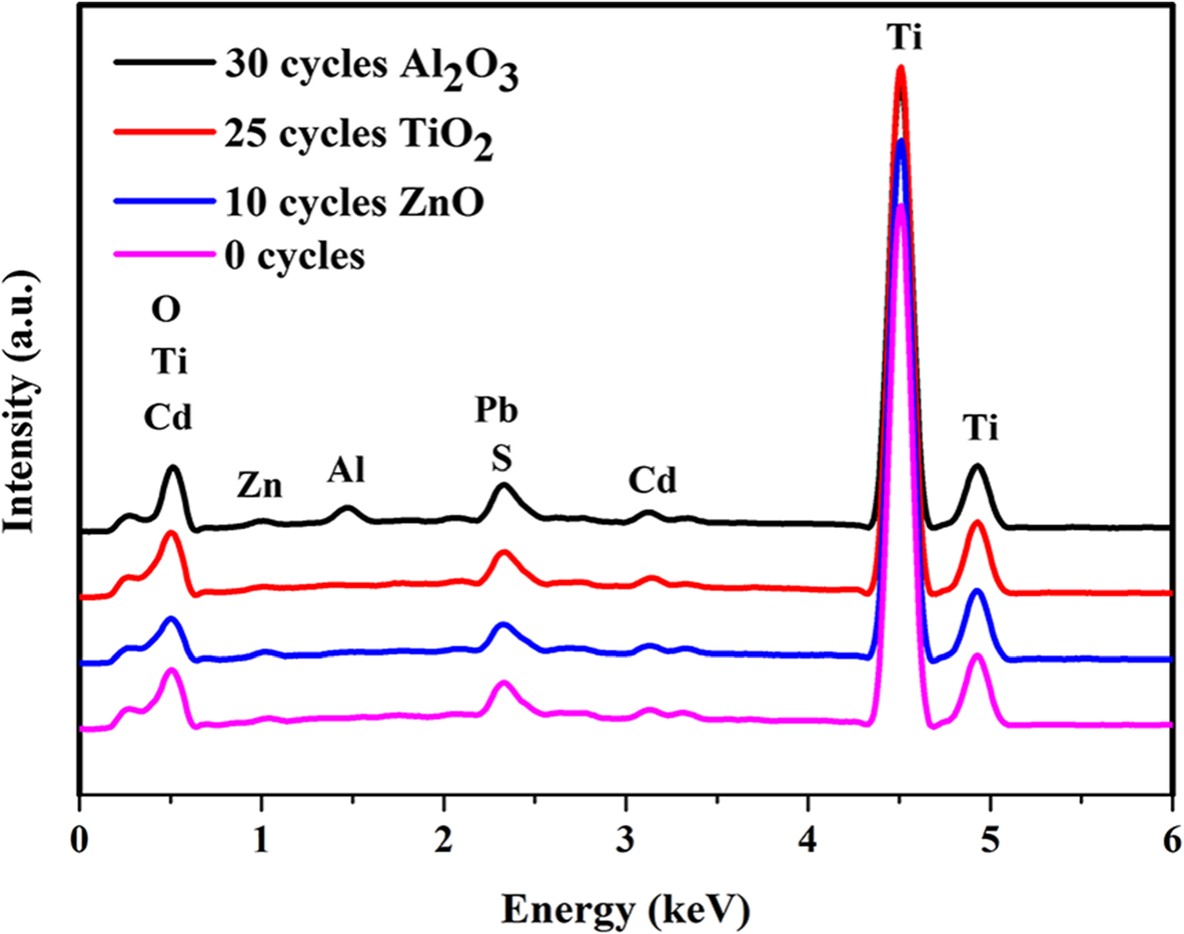
EDS results of the TNTAs/QDs, TNTAs/QDs/10 cycles ZnO, TNTAs/QDs/25 cycles TiO_2_, and TNTAs/QDs/30 cycles Al_2_O_3_ electrodes

In order to determine the crystal structure, the TNTAs/QDs, TNTAs/QDs/10 cycles ZnO, TNTAs/QDs/25 cycles TiO_2_, TNTAs/QDs/30 cycles Al_2_O_3_, electrodes were characterized by XRD, as shown in Fig. 3. The XRD pattern with the deposited passivation layers on the TNTAs/QDs is substantially coincidental, indicating that the passivation layers cannot change the crystal structure of the electrode. The peak of the corresponding oxides cannot be founded in the XRD patterns, but the previous TEM images in Fig. 2 and the EDS result shown in Fig. 4 support the presence of the oxide overlayer. This indicates amorphous structure of metal-oxide overlayer prepared by ALD. The diffraction peaks at 25.4°, 37.2°, 48.1°, 54.1°, and 55.2° correspond to the (101), (004), (200), (105), and (211) lattice planes of the TiO_2_ anatase phase (JCPDS No. 21-1272), respectively. The diffraction peaks of CdS, PbS, and ZnS are practically invisible in the four samples, most likely because the amount of the quantum dots are less than the XRD detection limitation.

The EDS pattern in Fig. 4 shows that Ti, O, Cd, Pb, Zn, and S elements are present in these four samples, indicating the presence of the CdS and PbS quantum dots on TNTAs. In addition, the Al element can be found in the pattern of the TNTAs/QDs/30 cycles Al_2_O_3_ sample. The atomic percentage of the Zn element in TNTAs/QDs/10 cycles ZnO is much greater than that of the other three samples, and the atomic percentage of the Ti element in the TNTAs/QDs/25 cycles TiO_2_ is also greater than that of the other three samples. These further suggest the presence of the passivation layers. The Na element in the TNTA/QD pattern may come from the precursor Na_2_S solution used in the preparation of the quantum dots. Atomic percentages of the elements derived from the EDS spectra are summarized in Table 1.

**Table 1:** Atomic percentages of the elements derived in EDS spectra

Photoelectrodes	O	Al	S	Ti	Zn	Cd	Pb
0 cycles [%]	48.75	0.00	1.08	48.37	0.22	0.44	1.13
30 cycles Al_2_O_3_ [%]	55.36	5.91	0.97	35.79	0.41	0.42	1.14
25 cycles TiO_2_ [%]	48.07	0.00	0.99	49.11	0.30	0.38	1.16
10 cycles ZnO [%]	48.25	0.00	1.06	48.11	0.81	0.39	1.38

**Fig. 5:**
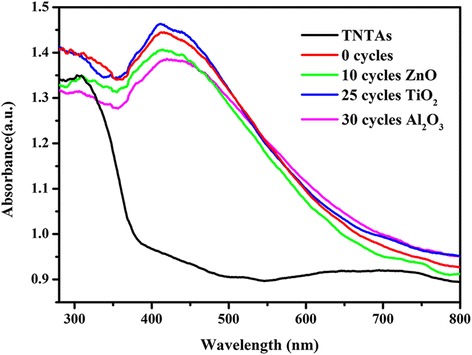
UV-vis absorption spectra of the pure TNTAs, TNTAs/QDs, TNTAs/QDs/10 cycles ZnO, TNTAs/QDs/25 cycles TiO_2_, and TNTAs/QDs/30 cycles Al_2_O_3_ electrodes

The UV-vis diffuse reflectance absorption spectra were characterized on these photoelectrodes, as shown in Fig. 5. The bandgap of the anatase type TiO_2_ is 3.2 eV, well corresponding to the absorption band edge measured at approximately 380 nm for the pure TNTAs. In contrast, the CdS and PbS quantum dot-co-sensitized TNTA photoelectrode shows a significant enhancement in a visible-light absorption range especially between 400 and 600 nm. The bandgap of CdS is 2.4 eV, which can only absorb light with wavelength less than 520 nm. After co-sensitization with PbS quantum dot, which has smaller bandgap (~0.4 eV), the absorption range can extend beyond 700 nm in the solar spectrum. At the same time, it is found that the deposition of the metal oxides on the TNTAs/QDs does not obviously change the light absorption properties, which suggests that the enhancement of the PEC performance observed later is not caused by the increase of the absorption of light by the passivation layer, similar to the results reported in other papers [32].

**Fig. 6:**
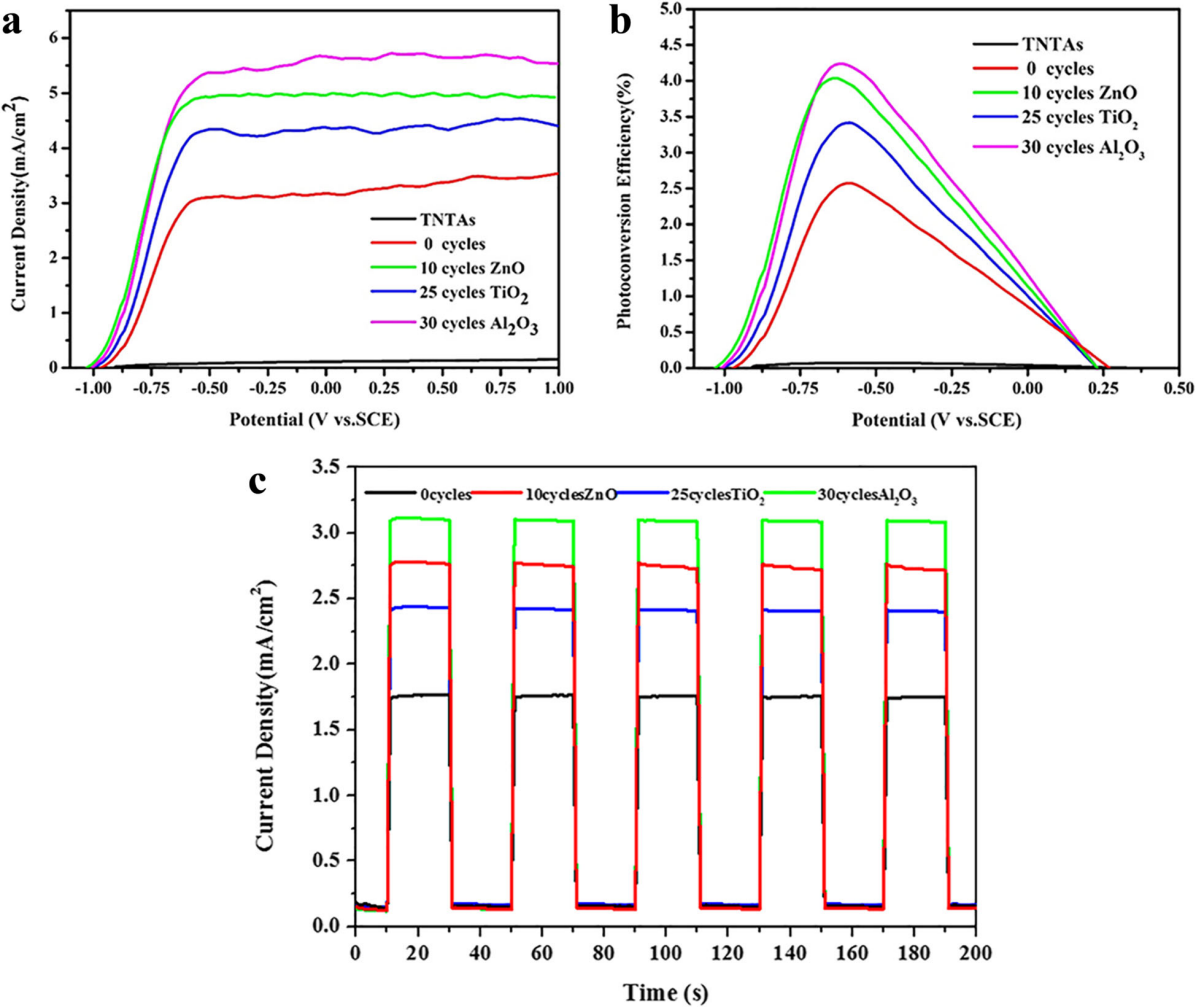
PEC properties of the TNTAs/QDs, TNTAs/QDs/10 cycles ZnO, TNTAs/QDs/25 cycles TiO_2_, and TNTAs/QDs/30 cycles Al_2_O_3_ electrodes with a surface area of 1 cm^2^. **a** LSV curves of the electrodes under simulated solar illumination. **b** Photoconversion efficiency derived from LSV curves. **c** Transient photoresponse under chopped light irradiation under a potential 0 V vs. SCE

The photoelectrochemical performance of the photoelectrodes with different cycle metal oxide passivation layers deposited on the TNTAs/QDs is shown in Fig. 6. Figure 6a is the LSV plots of the four electrodes, which is made negligible since the dark current density of all of the samples is less than 200 μA, which is negligible. Under the simulated solar irradiation, the photocurrent density of the pure TNTAs is 0.14 mA/cm^2^ at 1 V (vs. SCE). After quantum dot sensitization, the photocurrent density increases to 3.48 mA/cm^2^ under the same bias, which is 23 times higher than the photocurrent density of the unsensitized electrode. Under simulated solar illumination, the maximal photocurrent density of 10 cycles ZnO, 25 cycles TiO_2_, and 30 cycles Al_2_O_3_ overlayer coating on the TNTAs/QDs are 5.0, 4.3, and 5.6 mA/cm^2^, respectively. In comparison, photocurrent density of TNTAs/QDs/30 cycles Al_2_O_3_ is relatively maximal, which is 37 times higher than the photocurrent density of the TNTAs, and 1.6 times higher than the TNTAs/QDs. With the exception of TNTAs, the starting potentials of the photocurrent in all the samples are about −1.0 V (vs. SCE), indicating that the conduction band edge basically remains fixed.

The photoconversion efficiency of each electrode under different bias voltages is shown in Fig. 6b. The maximum photoconversion efficiencies of the TNTAs, TNTAs/QDs, TNTAs/QDs/10 cycles ZnO, TNTAs/QDs/25 cycles TiO_2_, and TNTAs/QDs/30 cycles Al_2_O_3_ electrodes are 0.06, 2.57, 3.95, 3.41, and 4.23% respectively. Thus, deposition of the metal-oxide layers onto the TNTA/QD photoelectrode can apparently increase their photoconversion efficiency. The efficiency of TNTAs/QDs/30 cycles Al_2_O_3_ is 1.6 times as much as TNTAs/QDs, better than the other photoelectrode. Compared with the TNTAs, the photoconversion efficiency could be maximally increased by 41 times after QDs co-sensitization and metal-oxide overlayer deposition.

Figure 6c presents the transient light response of the TNTA/QD electrodes with different ALD overlayers under the white-light excitation with the density of 80 mW/cm^2^ and the wavelength range of 565 ± 112 nm. The photocurrent of the four samples increased rapidly with the switching on of the LED lamp, which indicates that the photogenerated electrons in the electrode can rapidly be excited and injected into the TNTAs from the quantum dots. The photocurrent density of each electrode recorded after the five switch lamp cycles shows no significant decrease which suggests the stability of the electrodes.

**Fig. 7:**
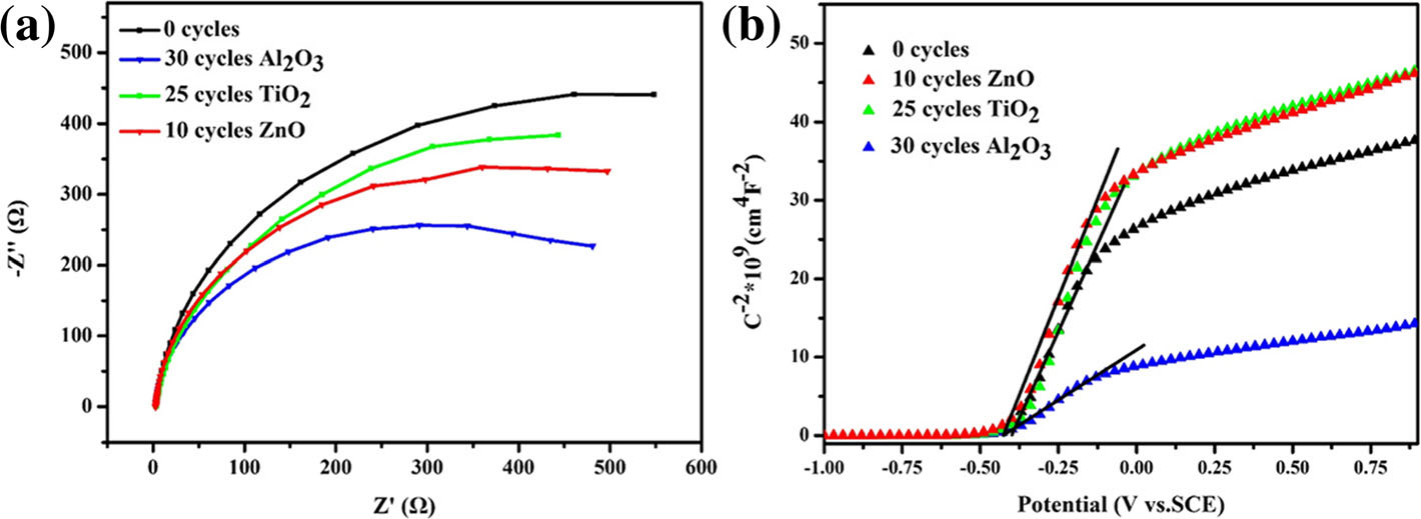
**a** EIS spectra of the TNTAs/QDs, TNTAs/QDs/10 cycles ZnO, TNTAs/QDs/25 cycles TiO_2_, and TNTAs/QDs/30 cycles Al_2_O_3_ electrodes. **b** Mott-Schottky analysis of the TNTAs/QDs, TNTAs/QDs/10 cycles ZnO, TNTAs/QDs/25 cycles TiO_2_, and TNTAs/QDs/30 cycles Al_2_O_3_ electrodes

The effects of the passivation layer on the photoelectric properties can be summarized as follows: (1) to passivate the surface defects of the TNTAs/quantum dots, (2) to play a catalytic effect in order to promote charge transfer, (3) to build a heterojunction to reduce the interface impedance. In order to clarify the underlying mechanism, these photoelectrodes were examined by EIS and Mott-Schottky measurements [30], and the results are shown in Fig. 7.

EIS results in Fig. 7a qualitatively demonstrate the charge transfer properties of the photoelectrodes with and without oxide passivation layer. The interfacial charge transfer impedance is inversely proportional to the arc diameters in the Nyquist plots. Among the four photoelectrodes, the interfacial charge transfer impedance increases as the following trend: TNTAs/QDs/30 cycles Al_2_O_3_, TNTAs/QDs/10 cycles ZnO, TNTAs/QDs/25 cycles TiO_2_, and TNTAs/QDs. It can be seen that all the oxide layers can reduce the interfacial charge transfer impedance, which will accelerate the carrier separation and transmission and then improve the photoelectrochemical performance. From the Mott-Schottky measurements in Fig. 7b, the carrier concentrations of the TNTAs/QDs, TNTAs/QDs/10 cycles ZnO, and TNTAs/QDs/25 cycles TiO_2_ electrodes could be calculated as 3.36 × 10^19^ cm^−3^, while the TNTAs/QDs/30 cycles Al_2_O_3_ electrode is 1.32 × 10^20^ cm^−3^. The charge carriers are generated in a semiconductor by the irradiation with light energy larger than its bandgap. There are a large number of defects on the surface of the TNTA/QD electrode. These defects work as recombination centers, leading to a decrease in carrier concentration. The Al_2_O_3_ layer coating on the surface of quantum dots plays a key role to passivate the surface defects and reduce charge recombination. Thus, Al_2_O_3_ coating can be found to increase the effective carrier concentration. In addition, when the electrode deposited with the Al_2_O_3_ passivation layer is exposed to light, the photogenerated holes in the valence band are rapidly injected from the quantum dots into the quantum dot/Al_2_O_3_ interface under the built-in electric field. Moreover, the negative charge in the Al_2_O_3_ layer would form a built-in electric field which can prevent the photogenerated electrons inside the bulk material, thus effectively reduce the recombination of photogenerated charges [30]. Although the surface passivation effect on TiO_2_ nanorod arrays with TiO_2_ coating were reported in the literature [23], according to our Mott-Schottky results, the carrier concentrations of the TNTA/QD electrode with TiO_2_ and ZnO coating keep the same as that of the TNTAs/QDs electrode, indicates that TiO_2_ and ZnO overlayers can hardly passivate the surface defects of the TNTAs/QDs electrode. The enhancement by TiO_2_ and ZnO overcoating is mainly due to the reduction of the interfacial charge transfer impedance.

The photochemical stability of the photoanodes is another important concern. After a series of electrochemical performance tests (transient photocurrent, LSV, and EIS tests) under illumination, there were no evident changes in photoelectrochemical performance for the three samples. But under 2 h continuous illumination, the photocurrent density of the photoelectrode with 30 cycles Al_2_O_3_ decayed slowly while that of the bare electrode suffered serious decay. This is because Al_2_O_3_ oxide layer has been partially dissolved in the electrolyte (a mixed solution of Na_2_SO_3_ and Na_2_S, used to prevent the oxidation of sulfide ions), leading to decrease of the transformation of photogenerated electrons and holes. The photochemical stability of the photoelectrodes with the ZnO and TiO_2_ overlayers shows similar decay to that with Al_2_O_3_, which is possibly due to their similar amorphous nature. In terms of long-term photochemical stability, future work is required to understand the underlying reason and to optimize the overlayer by improving the crystallinity or combining different oxide layers by ALD.

## Conclusions

ALD technology has been applied to deposit various uniform and compact oxide overlayers, Al_2_O_3_, TiO_2_, and ZnO with controllable thickness, on the TNTAs/QDs structure. These overlayers within the tunneling thickness of approximately 1.5 nm can enhance the photoelectric properties of the TNTA/QD photoelectrodes. Under simulated sunlight, the maximal photocurrent densities of the TNTAs/QDs with 10 cycles ZnO, 25 cycles TiO_2_, and 30 cycles Al_2_O_3_ overlayers are 5.0, 4.3, and 5.6 mA/cm^2^, respectively. All the three oxide overcoating can improve the photoelectrochemical performance of the TNTAs/QDs, but the underlying mechanisms are different. Al_2_O_3_ overlayer can chemically passivate the surface defects of the TNTA/QDs electrode, reduce the charge recombination, and improve the transfer efficiency of the charge in the bulk material and the transfer kinetics at the interface. This leads to the superior performance than TiO_2_ and ZnO overlayers, which just reduce the interfacial charge transfer impedance in varying degrees without increasing the carrier concentration. This work demonstrates that the proper selection of metal-oxide overlayer is of great importance to improve the photoelectric properties, and the ALD technology is a promising and facile method for surface modification to prepare the high-performance electrodes in the photoelectrochemical applications.
